# Effective Dose Reduction of Emamectin Benzoate Through Inhibition of *Bx-SDR3* in Pine Wood Nematode Management

**DOI:** 10.3390/ijms26041679

**Published:** 2025-02-16

**Authors:** Yuting Zhuang, Rui Xia, Fan Yang, Zhao Xu, Guanjun Liang, Ruizhi Zhang, Yue Bao, Feng Wang

**Affiliations:** 1 Key Laboratory of Alien Forest Pest Detection and Control-Heilongjiang Province, Northeast Forestry University of Forestry, Harbin 150040, China; 2022120162@nefu.edu.cn (Y.Z.); xiarui@nefu.edu.cn (R.X.); 18845725074@163.com (F.Y.); xuzhao976@gmail.com (Z.X.); 15035243@nefu.edu.cn (G.L.); zhangruizhi@nefu.edu.cn (R.Z.); baoyue5717@163.com (Y.B.); 2 Key Laboratory of National Forestry and Grassland Administration on Northeast Area Forest and Grass Dangerous Peat Management and Control, Shenyang Institute of Technology, Shenyang 113122, China; 3 State Key Laboratory of Tree Genetics and Breeding, Northeast Forestry University of Forestry, Harbin 150040, China

**Keywords:** *Bursaphelenchus xylophilus*, *Pinus koraiensis*, Emamectin benzoate, Biocontrol, Detoxification mechanisms, RNAi

## Abstract

Pine wood nematodes (*Bursaphelenchus xylophilus*, PWNs) are a major threat to *Pinus koraiensis* in northeast China, and emamectin benzoate (EB) is commonly used for their control. Although high doses of EB can alleviate symptoms of pine wilt disease (PWD), they do not fully eradicate PWNs due to their detoxification mechanisms. This study investigates the content of EB in *P. koraiensis* and its efficacy in controlling PWNs after exogenous application of EB. We found that while EB significantly reduced PWN populations, it did not eliminate them. Transcriptomic analysis of PWNs treated with concentration at 20% (LC_20_) revealed that PWNs exhibit detoxification responses to low EB concentrations (LC_20_), driven by the *Bx-SDR3* gene. RNA interference (RNAi)-mediated silencing of this gene decreased the detoxification ability of PWNs and enhanced the toxic effects of LC_20_ EB by 20.9%. These results highlight the key role of *Bx-SDR3* in PWN detoxification and suggest that targeting this gene could improve the effectiveness of EB, offering a promising strategy for more efficient and eco-friendly pest management.

## 1. Introduction

Pine wilt disease (PWD), caused by the *Bursaphelenchus xylophilus* (pine wood nematode, PWN), is a major forest disease that requires quarantine measures [1,2]. The migration of PWNs from North America to East Asia has resulted in significant damage to coniferous tree species across multiple countries [3]. PWN infestation has impacted 663 counties across 18 provinces (cities) [4]. *Pinus koraiensis*, a commercially important tree species native to northeast China, was first reported with PWN infection in Gwangju, South Korea, in 2006 [5]. With the ongoing northward spread of PWNs, we first identified naturally infected *P. koraiensis* in Dandong City, Liaoning Province, China, in 2017 [6].

With the growing severity of PWN infection in *P. koraiensis* and the limitations of existing control methods, exploring more sustainable alternatives became essential. Emamectin benzoate (EB) is a semi-synthetic derivative of avermectin. It has been modified to enhance its stability and effectiveness, particularly in agricultural and forestry applications. EB has been demonstrated to activate chloride channels that are controlled by glutamate in PWN muscle cells [7,8,9], influencing neurotransmitter processes through ligand binding and thereby exerting a toxic effect on PWN. Therefore, EB was approved to control PWN for a considerable period of time. Furthermore, a study conducted in 2023 demonstrated that the administration of *P. thunbergii* in an EB emulsion resulted in a significant reduction in the incidence of PWD, with efficacy lasting for a minimum of 18 months [10]. However, during the process of applying EB to control PWN, we found that some PWNs exposed to EB could still survive [11]. The reduced efficacy of EB against PWNs may be attributed to their detoxification function, which diminishes the nematicide’s toxicity [9]. If the genes associated with detoxification are identified and silenced, it may be possible to eradicate the PWN using low doses of nematicides.

Short-chain dehydrogenases/reductases (SDRs) are a widely present class of enzymes in organisms. They constitute a large family of NAD(P)(H)-dependent oxidoreductases, sharing sequence motifs and similar mechanisms. SDRs, found in nearly all organisms, play crucial metabolic roles, including detoxification and regulation of signaling molecules [12,13]. In 2019, it was demonstrated that SDRs reduce toxic aldehydes by catalyzing their conversion into less harmful and easily excreted products, maintaining low aldehyde levels in the body [14]. Studies have shown that increased expression of *SDR* genes correlates with enhanced drug resistance at most stages of *Haemonchus contortus* [15]. It seems reasonable to posit that the *SDR* gene could serve as an effective RNA interference (RNAi) target to enhance the efficacy of nematicides through gene silencing.

The aim of this study was to explore the content of EB in *P. koraiensis* and its efficacy in controlling PWNs after exogenous application, and the impact of EB on PWN populations and the underlying molecular mechanisms of PWN responses to EB. We also explored the potential of enhancing the efficacy of EB by targeting a key gene involved in PWN detoxification. Our research focused on determining the effect of EB on PWN populations, conducting transcriptomic analysis of PWNs treated with a low concentration of EB (LC_20_) to identify genes associated with detoxification, and using RNAi to silence a key detoxification gene, *Bx-SDR3*, to assess its impact on the toxicity of EB to PWNs. The significance of this study lies in its contribution to a better understanding of the interaction between EB and PWNs. By identifying the key role of *Bx-SDR3* in PWN detoxification, our findings provide a novel strategy for improving the effectiveness of EB in controlling PWNs. This approach not only offers a more efficient pest management method but also has the potential to reduce the environmental impact associated with high-dose pesticide applications, thus contributing to more sustainable and eco-friendly forest protection.

## 2. Results

### 2.1. Efficacy of EB in Reducing PWN Populations in P. koraiensis

**Figure 1:**
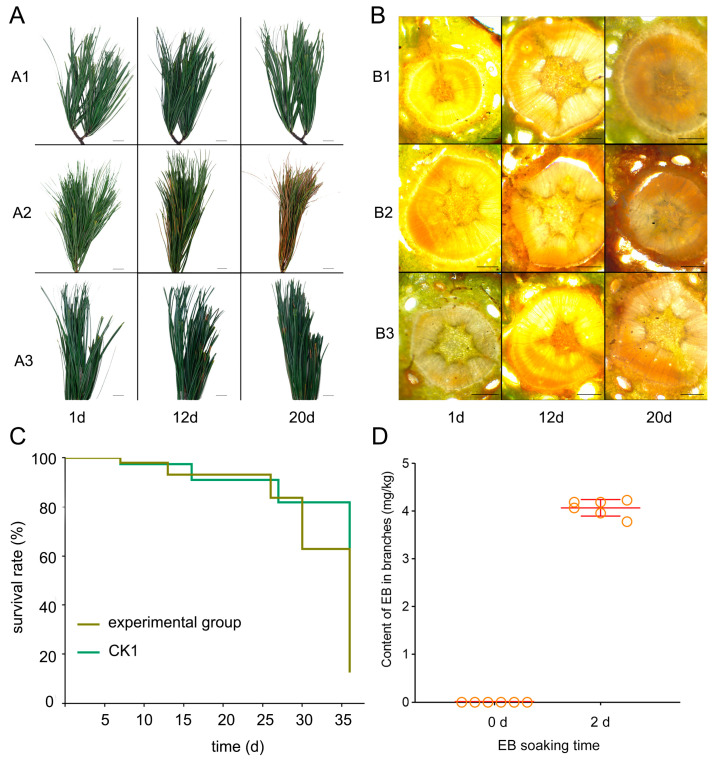
Illustrates the alteration of symptoms on the vulnerable *P. koraiensis* and the variations in activity of PWNs following the application of a diluted quantity of EB. (**A**) Manifestations of *P. koraiensis* branches: (**A1**), symptoms observed in the branches of CK2; (**A2**), symptoms observed in the branches of CK1; (**A3**), symptoms observed in the branches of the experimental group, scale bar: 25 mm. (**B**) Symptoms observed in the cross-section of *P. koraiensis* stems: (**B1**), symptoms observed in the stem cross-section of CK2; (**B2**), symptoms observed in the stem cross-section of CK1; (**B3**), symptoms observed in the stem cross-section of the experimental group, scale bar: 500 μm. (**C**) Survival curve of PWNs following a 24 h treatment with a 60 mg/L concentration of EB. (**D**) EB content in *P. koraiensis* branches after soaking for 2 d. The orange ring represents the content of EB in the branches. A total of 6 repetitions, and made the error line.

We continuously observed the differences in symptoms between EB-treated and untreated *P. koraiensis* branches inoculated by PWNs to evaluate the therapeutic effect of EB on PWD. The results of continuous observation showed that the branches of *P. koraiensis* treated with ddH_2_O in control group 2 (CK2) remained healthy (Figure 1(A1,B1)). On the 12 d, the needles of *P. koraiensis* of the control group 1 (CK1) (inoculated with PWNs) showed symptoms of chlorosis. By 20 d, almost all needles became yellow (Figure 1(A2,B2)). When *P. koraiensis* branches were treated with a 60 mg/L concentration of EB after being inoculated with PWNs, there was a notable decrease in disease severity compared to branches that were only inoculated with PWNs (Figure 1(A3,B3)). The above results show that EB has a significant therapeutic effect on *P. koraiensis* inoculated with PWNs.

For detecting the effectiveness of EB in controlling PWNs, we reisolated the live PWNs in the *P. koraiensis* branches. The results showed that with the increase of drug concentration, the number of PWNs isolated gradually decreased, but live PWNs could always be isolated. The application of a 60 mg/L EB solution resulted in a significant reduction in nematode numbers compared to the control. The treated group had 600 to 800 fewer nematodes compared to CK1 (Figure 1C). To further investigate the EB content in the branches of *P. koraiensis* following a two-day immersion, we quantified the EB concentration using targeted metabolism. The concentration of EB within the branches of *P. koraiensis* was determined to be 4.07 mg/kg (Figure 1D); however, the concentration within the tree was only 6.7% of the original EB solution. The findings indicated that while there was an alleviation of PWD symptoms and a reduction in the total population of PWN, the treatment did not eradicate PWN.

### 2.2. Elevated Concentrations of EB Are Required for the Effective Poisoning of PWNs

Following the evaluation of *P. koraiensis*’s symptom response to EB treatment, we conducted a toxicity assessment to determine EB’s effectiveness against PWNs. The results indicated that poisoning PWNs necessitates high EB concentrations, whereas lower doses merely induce a pseudo-death state.

**Figure 2:**
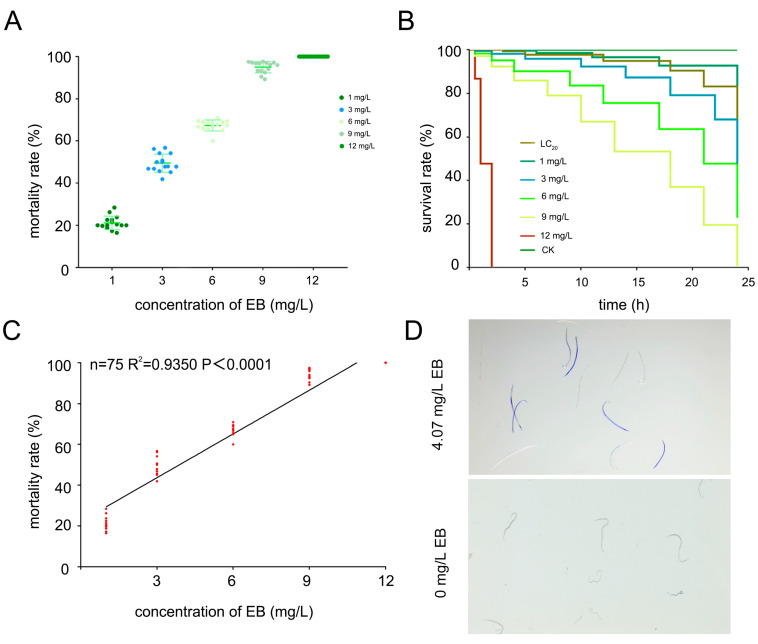
Effect of EB on the survival of PWD. (**A**) The deleterious impact of EB concentrations at various time points on PWNs over a 24 h period. (**B**) The survival curve of PWNs within 24 h following treatment with EB at varying concentrations. (**C**) The correlation between the mortality rate of PWNs and the concentration of EB. (**D**) Methyl blue staining of PWNs in 0 mg/L EB treatment group and 4.07 mg/L EB treatment group.

The mortality of PWNs treated with different concentrations of EB for 24 h was calculated. At an EB concentration of 12 mg/L, all PWN individuals died. The mortality rate was (49.48 ± 0.041)% at 3 mg/L and was (21.19 ± 0.031)% at 1 mg/L (Figure 2A,B, Table 1); these results are presented in Table 1. Based on the mortality rate at five concentrations, an independent regression equation of EB was established (Table 1). The data indicated a positive correlation between EB concentration and PWN mortality at concentrations between 1 mg/L and 12 mg/L (Figure 2C). Based on the independent regression equation of EB, the half lethal concentration (LC_50_) value was determined to be 3.61 mg/L, and the LC_20_ value was found to be 1.59 mg/L. The EB concentration in the tree was 4.07 mg/L, so PWNs were treated at this concentration. With methyl blue staining, it was found that the dead PWNs were stained blue (Figure 2D), which made it easier to distinguish dead PWNs from live PWNs.

### 2.3. Transcriptomic Analysis Reveals Bx-SDR3’s Role in PWN Response to Low-Dose EB Exposure

**Figure 3:**
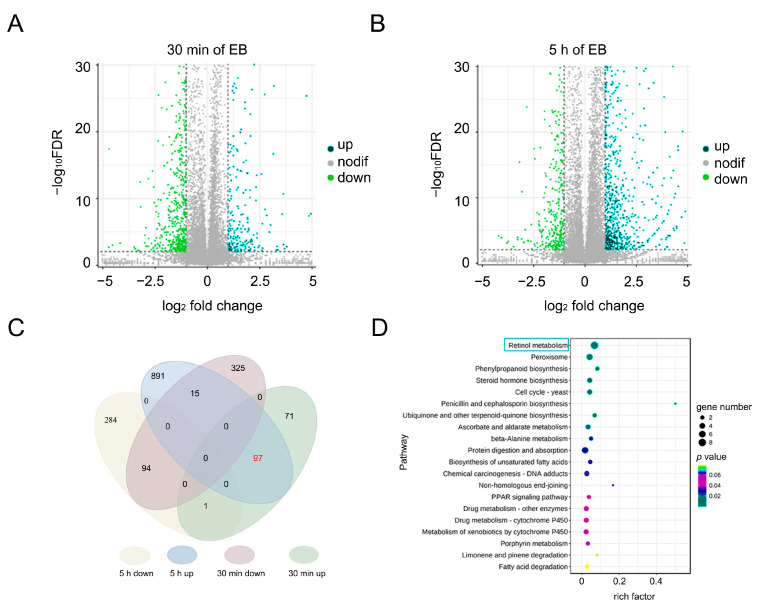
Analysis of transcriptome data of PWNs after 30 min and 5 h of EB treatment. (**A**) The gene expression profile of PWNs subjected to LC_20_ EB treatment for 30 min. (**B**) Profile of gene expression in PWNs subjected to LC_20_ EB treatment for 5 h. (**C**) Evaluation of differentially expressed genes in PWNs after 30 min and 5 h of LC_20_ EB treatment. The 97 red-tagged genes were up-regulated at 30 min and 5 h after EB treatment. (**D**) Analysis of KEGG pathway enrichment for differentially expressed genes.

To elucidate the mechanisms enabling PWN to endure low-dose conditions, we conducted a transcriptomic analysis to investigate the expression changes in genes associated with EB detoxification following treatment. The investigation employed the PossionDis method to detect differential gene expression. The expression patterns of six differentially expressed genes were verified by RT-qPCR. The results showed that the RT-qPCR of six genes was consistent with the results of transcriptome sequencing (Figure S1), indicating that the transcriptome data were credible. After a 30 min exposure to LC_20_ EB, the PWNs transcriptome identified 612 genes that were differently expressed, with 178 genes showing increased expression and 434 genes showing decreased expression (Figure 3A). After subjecting the samples to a prolonged treatment of 5 h with LC_20_ EB, a total of 1428 genes showed differential expression, with 1049 genes upregulated and 339 genes downregulated (Figure 3B). Significantly, out of these, a total of 97 genes exhibited consistent upregulation in response to both the 30 min and 5 h treatments with LC_20_ EB. This highlights a core set of genes that are highly responsive to EB exposure over time (Figure 3C).

Kyoto Encyclopedia of Genes and Genomes (KEGG) Pathway enrichment analysis of the 97 upregulated genes revealed that 8 were involved in the retinol metabolism pathway (ko00830). Examination of these eight genes revealed that four belonged to the short-chain dehydrogenases/reductases family. This suggested that these genes may have a role in the response of PWN to EB exposure (Figure 3D). The results of transcriptomic analysis indicated that *Bx-SDR* genes were significantly responsive to EB exposure over time. The correlation analysis between the four *Bx-SDR* genes and the survival rate of PWNs showed that the *Bx-SDR3* gene was significantly positively correlated with the survival rate of PWNs, so the *Bx-SDR3* gene was selected for research.

### 2.4. Identification and Analysis of Functional Diversity of Bx-SDR3 Through Bioinformatics

**Figure 4:**
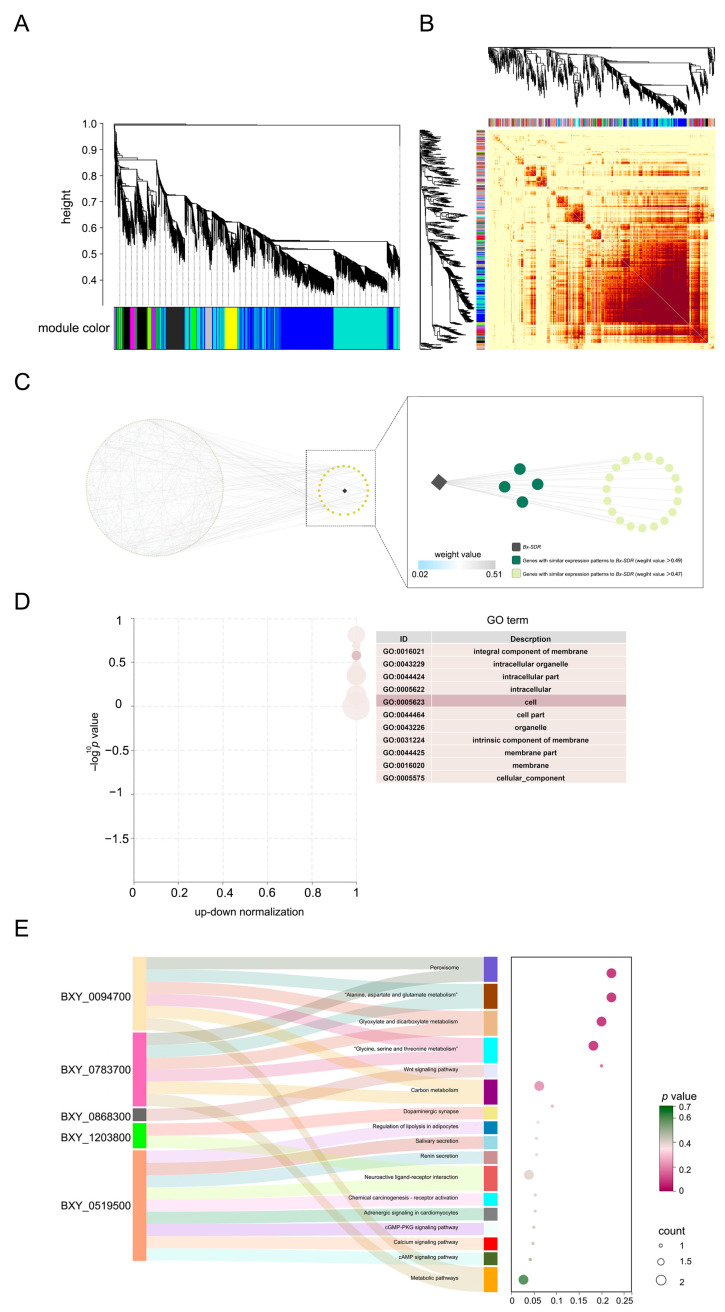
Evaluation of functionally similar genes to *Bx-SDR3* via WGCNA. (**A**) Tree of dynamic segmentation for acquiring gene modules. (**B**) Thermal map of WGCNA. (**C**) The network map of the lightcyan module. (**D**) Enrichment analysis of gene GO in lightcyan module. (**E**) Enrichment analysis of KEGG gene in lightcyan module.

To find genes related to *Bx-SDR3*, the weighted gene co-expression network analysis (WGCNA) was selected for analysis. Through the utilization of WGCNA, a total of 98 unique gene co-expression modules were identified. The relationships between these modules were visualized with a heatmap, where each branch of the dendrogram represents clusters of genes that are strongly associated with one another (Figure 4A). A significant consistency in gene expression patterns was discovered within distinct modules. Significantly, the heatmap displays bright blocks along the diagonal, indicating increased topological overlap and closer linkages between genes inside the same module (Figure 4B).

*Bx-SDR3* belongs to the lightcyan module, in which 26 genes were identified that have similar expression patterns to *Bx-SDR3*. Each of these genes had a weight value greater than 0.47. Four genes (BXY_0801300, BXY_1065900, BXY_1011500, and BXY_0268100) were identified as being related to *Bx-SDR3*. BXY_0801300 is the gene that encodes the Niemann–Pick C1 protein, BXY_1065900 encodes Cuticlin-1, BXY_1011500 encodes collagen (type IV, alpha), and BXY_0268100 is the gene that encodes mucin-2. These genes had weight values above 0.49, suggesting their potential importance. Further inquiry was warranted to explore their potential (Figure 4C).

To uncover the functional roles of *Bx-SDR3* related genes, we employed Gene Ontology (GO) and KEGG enrichment analysis on the 243 genes in the lightcyan module. The results showed that these genes were primarily involved in various metabolic activities, indicating a wide range of functions and interactions within this module. The KEGG enrichment analysis revealed that the *Bx-SDR3* related genes play important roles in crucial biological processes and metabolic pathways.

An analysis of GO enrichment was conducted on the 243 genes in the lightcyan module (Figure 4D). The top 25 terms with the greatest gene counts were picked for the categories of cellular component, molecular function, and biological process. These genes mostly contributed to a range of metabolic activities, as indicated by their involvement in GO terms such as GO: 0044265, GO: 0044248, GO: 0044260, GO: 1901575, GO: 0009056, and GO: 0043170. The presence of a subset of genes was involved in the organization of cellular components (GO: 0044085, GO: 0044087, GO: 0071840), while other genes were related with transferase activities (GO: 0016747, GO: 0016746, GO: 0016740), suggesting that this module had a wide range of functions and interactions within.

SDR is one of the 243 genes in the lightcyan module. Using WGCNA to identify 26 genes with similar expression patterns can help us better understand the function and role of SDR gene in the detoxification of EB by PWN. GO and KEGG enrichment analysis of the 243 genes in the lightcyan module revealed their involvement in 20 pathways, including those related to *Bx-SDR3* (Figure 4E). The pathways related to *Bx-SDR3* are mainly enriched in pathways related to metabolic processes: alanine, aspartate, and glutamate (Ala-Asp-Glu) metabolism (ko00250), peroxisome (ko04146), glyoxylate, and dicarboxylate metabolism (ko00630), methane metabolism (ko00680), glycine, serine, and threonine (Gly-Ser-Thr) metabolism (ko00260), Wnt signaling pathway (ko04310), biosynthesis of secondary metabolites (ko01110), microbial metabolism in different environments (ko01120), and carbon metabolism (ko01200). The aforementioned metabolic pathways facilitate the detoxification process through various mechanisms, including the direct degradation of toxic substances, the generation of intermediate products necessary for detoxification, and the protection of cells from toxic damage. Each pathway can contribute to detoxification under specific biological and environmental conditions.

### 2.5. Enhancing EB Efficacy in PWN Management by Inhibiting Bx-SDR3 for Effective Dose Reduction

**Figure 5:**
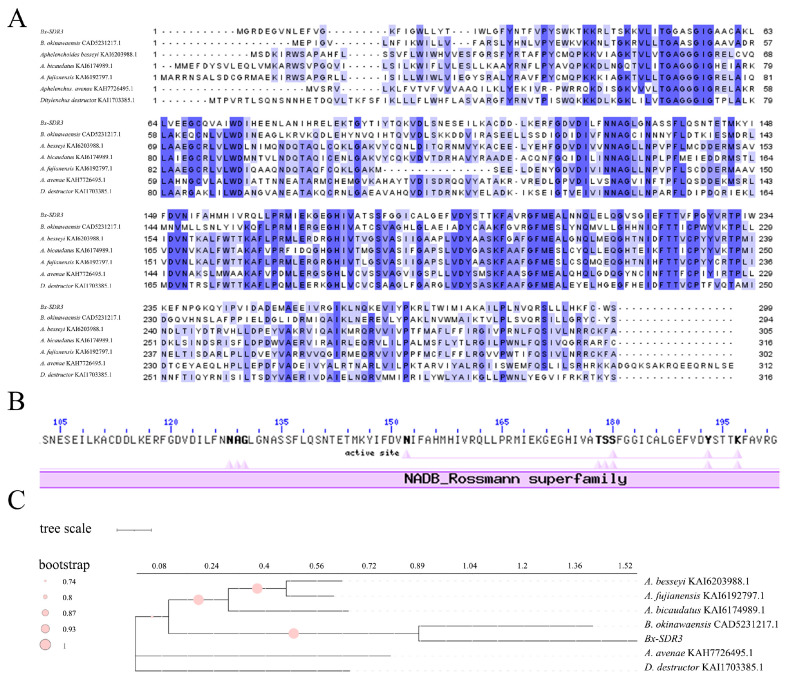
*Bx-SDR3* bioinformatics analysis. (**A**) *Bx-SDR3* sequence alignment. The blue color intensity of a site reflects its conservation degree: the darkest shade indicates complete identity across all species at that site (highly conserved), while lighter shades suggest increasing divergence among species and a progressive reduction in conservation. (**B**) Conserved domains of *Bx-SDR3*. (**C**) The maximum likelihood tree of *Bx-SDR3*.

To identify the *Bx-SDR3* gene sequence, the *Bx-SDR3* gene was cloned and subjected to bioinformatics analysis. Sequencing results showed that the *Bx-SDR3* gene is 900 bp in length (Figure S2) and encodes a 299-amino acid protein, confirmed through Open reading frame (ORF) analysis. The protein’s isoelectric point was determined to be 6.78, and it had a molecular mass of 33,850 Da. Six homologous sequences were selected from the National Center for Biotechnology Information (NCBI) database (Figure 5A), and the conserved domain analysis confirmed the presence of the NADB Rossmann superfamily domain in all of them (Figure 5B). The phylogenetic analysis of six species revealed that *Bx-SDR3* does not align directly with any SDR proteins from other plant-parasitic nematodes. This indicates that *Bx-SDR3* represented a separate evolutionary lineage (Figure 5C).

**Figure 6:**
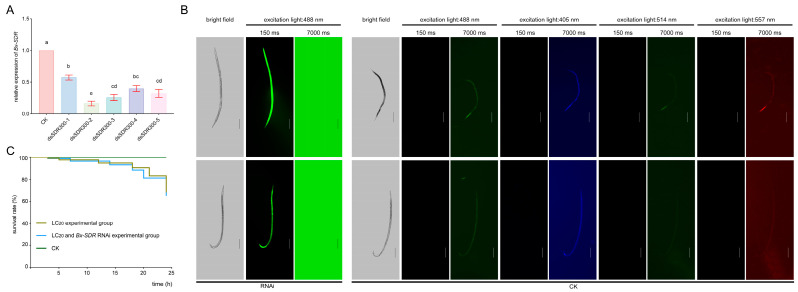
Enhancing EB efficacy by inhibiting *Bx-SDR3*. (**A**) Changes in the relative expression levels of *Bx-SDR3* under different RNAi targets. Letter a: the maximum average number marked with the letter a. Letter b: The maximum average is compared with the following averages. Where the difference is not significant, the letter a is marked until a significant difference is marked with the letter b Letter labeling followed by analogy. Where there is an identically marked letter, the difference is not significant; where there are different marked letters, the difference is significant. (**B**) Photo images of PWN after *Bx-SDR3* RNAi marked by FAM (black background), scale bar: 100 μm. CK, autofluorescence of male and female PWN under different wavelengths of excitation light. (**C**) Survival curve of PWNs treated with LC_20_ EB after RNAi within 24 h.

According to the *Bx-SDR3* and homologous sequence, five RNAi target identifications for *Bx-SDR3* gene silencing (dsSDR300-1, dsSDR300-2, dsSDR300-3, dsSDR300-4, and dsSDR300-5) were selected. To assess the RNAi efficiency of different dsRNA segments on FSBx, 10,000 FSBx were soaked in 2 mg/mL dsRNA synthesized in vitro, and 10,000 FSBx soaked in M9 Buffer were used as control. After soaking for 24 h, *Bx-SDR3* gene expression was verified by RT-qPCR. Compared to the control group, the *Bx-SDR3* gene expression in the RNAi-treated FSBx was significantly down-regulated by 42.67% (dsSDR300-1), 83.67% (dsSDR300-2), 74.33% (dsSDR300-3), 60% (dsSDR300-4), and 67.67% (dsSDR300-5). These results suggest that different regions of dsRNA all produce RNAi effect in FSBx and decrease the expression level of *Bx-SDR3* gene in PWN, among which dsSDR300-2 has the best effect; therefore, dsSDR300-2 was selected for RNAi to further verify the gene function (Figure 6A).

After soaking, the PWNs displayed green fluorescence, confirming successful dsRNA absorption. To avoid the effect of autofluorescence on RNAi fluorescence, we photographed the autofluorescence of PWNs at four excitation wavelengths (405 nm, 488 nm, 514 nm, and 557 nm). It was found that there was a significant difference between autofluorescence and RNAi fluorescence at exposure time of 150 ms and 7000 ms, indicating that RNAi had been successful (Figure 6B). In toxicity assays utilizing LC_20_ EB, the post-RNAi *Bx-SDR3* exhibited an increased mortality rate (Figure 6C). The expression level changes of *Bx-SDR3* and functionally similar genes in PWNs were confirmed after 24 h of exposure to different EB dosages using RT-qPCR. The results showed that the expression levels of *Bx-SDR3* and its associated gene rose within 24 h of EB treatment at all concentrations, consistent with the findings of transcriptome sequencing. Significantly, after RNAi, the relative expression level of *Bx-SDR3* decreased (Figure 6C). The survival rate of nematodes following RNAi treatment with dsSDR300-2 at a low concentration of EB (1.59 mg/L) was 63.33%, representing a reduction of 20.9% compared to the non-RNAi control group.

To reveal the mechanism of PWN survival in the low doses of EB, as well as to find a technical method of effective dose reduction of EB in PWN management, we employed RNAi technology to target and silence the detoxification gene *Bx-SDR3*. Results showed that RNAi-mediated silencing of *Bx-SDR3* significantly reduced PWN survival rates at low EB concentrations. This finding highlighted the potential of targeting *Bx-SDR3* for enhancing the efficacy of EB in managing PWN, offering novel insights for developing more effective biological control strategies.

## 3. Discussion

To identify a technical approach for reducing EB doses in PWN management, RNAi technology was employed to target and silence the detoxification gene *Bx-SDR3*. Results showed that RNAi-mediated silencing of *Bx-SDR3* significantly reduced PWN survival rates at low EB concentrations. By applying EB to inoculated *P. koraiensis* branches, the lowest and highest EB doses were determined to reduce the symptoms of PWN and the toxicity to *P. koraiensis*. The doses of LC_50_ of PWN were determined by nematode toxicity test. Bioinformatics analyses, including transcriptome and WGCNA, identified the *Bx-SDR3* gene as being associated with EB exposure. The target site sequence for RNAi of *Bx-SDR3* has been cloned and identified. After RNAi silencing of *Bx-SDR3* gene, its LC_50_ decreased to 3.15 mg/L. This indicates that *Bx-SDR3* functions in the detoxification of EB. This result also demonstrates that EB dose reduction can be achieved through inhibition of *Bx-SDR3* in PWN management.

### 3.1. Environmentally Hazardous PWN Chemical Control Methods

Chemical control is the primary method for managing PWD, including fumigating wooden packaging materials, pine wood chips, and trunk injections [16]. Traditional nematode control methods, such as methyl bromide and phosphine, have strong toxicity while their ability to kill parasitic nematodes gradually decreases, which brings great risks to the environment in order to kill nematodes. Therefore, as an important field for controlling PWN, it is crucial to determine efficient, low toxicity, and environmentally friendly treatment methods. Given the high toxicity and declining efficacy of traditional chemical treatments like methyl bromide and phosphine, alongside their environmental risks, chemical control methods are increasingly unsustainable for managing PWN [17].

### 3.2. EB Needs to Improve Its Efficacy to Be More Effective in PWN Control

To address the shortcomings of traditional chemical control methods, EB has emerged as a promising alternative, offering both strong nematicidal effects and environmental safety. It activates glutamate-gated chloride ion channels in neuromuscular cells [11]. The main mechanism of action of EB involves the activation of glutamate-gated chloride channels (GluCls). This activation causes an influx of chloride ions, leading to excessive polarization of nematode nerve cells, ultimately resulting in paralysis and death [9]. In 2000, the effectiveness of EB as a candidate for tree trunk injection was determined by measuring the inhibitory concentration (IC) value against PWN. Research demonstrated that EB has a significant inhibitory effect on PWNs, indicating its potential application in preventing and controlling PWD [18]. In this context, EB exhibits strong nematicidal activity and low toxicity, and its long-term environmental impact is minimal, making it a preferred choice for controlling PWN and an environmentally friendly option [19].

However, while EB shows great potential, its inability to effectively kill all nematodes at environmentally safe concentrations necessitates the use of higher doses to achieve satisfactory results, highlighting an urgent scientific challenge that requires resolution. In our experiment, the mortality rate of 12 mg/L EB to PWNs is 100%, and the mortality rate of 1 mg/L EB to PWNs was 10%. The LC_50_ EB of EB to PWN was 3.61 mg/L, and the LC_20_ of EB to PWNs was 1.59 mg/L. It showed that the effectiveness of EB against PWN is based on its concentration. There was a positive correlation between the concentration of EB and the mortality rate of PWN. Through optimal screening, it was shown that a concentration of 60 mg/L of EB was not harmful to *P. koraiensis* branches, but was highly successful in removing PWNs. The toxicity of EB to PWN varied with concentration. The effective concentration of EB in the *P. koraiensis* branch was determined to be 4.07 mg/kg. This concentration could kill the PWN in a short period of time, effectively reducing its population and acting as a PWD control.

### 3.3. Suppressing the Bx-SDR Gene Can Enhance PWN Control by EB

The reduced efficacy of EB in poisoning nematodes is due to detoxification mechanisms in PWN, with *Bx-SDR* playing a key role in this process. Suppressing *Bx-SDR* gene function has been shown to significantly enhance the effectiveness of EB in controlling PWN populations [20]. In this study, the correlation between the expression of the four *Bx-SDR* genes and the survival rate of PWNs was analyzed. *Bx-SDR3* was selected, but the other three *Bx-SDR* genes were also up-regulated in the detoxification process of PWNs, and their functions will be further studied in the future. In 2023, by identifying members of the SDR family in the *H. contortus* genome and comparing them with SDRs in *Caenorhabditis elegans* and *Ovis aries* (a typical host of *H. contortus*), research has found that *SDR1*, *SDR12*, *SDR13*, and *SDR16* may be candidate genes for drug resistance. These SDRs show increased expression in most stages of *H. contortus* [15].

In *C. elegans*, its detoxification pathway consists of two stages, Phase I enzymes (cytochrome P450, SDR) and Phase II enzymes (UDP-glucuronosyltransferase, glutathione S-transferase), which can alter dangerous chemicals [21]. After administration of Albendazole therapy, the SDR activity of *C. elegans* was significantly increased, highlighting the involvement of this enzyme in the response to xenobiotic stress [22]. However, compared with other nematodes that feed on plants, PWN has more detoxification enzymes, which helps them survive in nematicides. Research indicates that when host plants are infected by PWN, the defense substances released by the host plant may stimulate the growth of gene groups related to PWN’s heterotrophic detoxification pathway, hence enhancing the survival of nematodes. Enzymes such as flavin-containing monooxygenase (FMO), CYP450, and SDR are crucial in this process [23].

The findings of this study are of critical importance, as they reveal a novel genetic target for improving chemical control strategies against PWN. By targeting *Bx-SDR3*, we can reduce the required concentrations of EB, thereby minimizing environmental impact while enhancing nematode mortality. This research provides a valuable foundation for future work in developing more sustainable and effective approaches to PWD management, offering promising avenues for integrating genetic and chemical control methods to achieve better outcomes in forestry and environmental conservation efforts.

### 3.4. Potential and Challenges of RNAi-Based Strategies for Enhancing EB Efficacy in PWNs Control

In recent years, RNAi has emerged as a promising strategy for pest control, offering a targeted and environmentally sustainable approach to managing harmful organisms such as PWN [24]. In this study, we utilized RNAi to silence the *Bx-SDR3* gene, which is implicated in the detoxification of EB by PWNs. Our results demonstrate that RNAi-mediated silencing of *Bx-SDR3* significantly enhanced the toxicity of EB, reducing PWN populations by 20.9% more than with EB treatment alone. This suggests that RNAi can effectively disrupt the detoxification mechanisms of PWNs, potentially overcoming one of the major barriers to the successful application of EB in PWNs management.

However, while RNAi holds promise, several challenges remain for its practical implementation [25]. First, PWN is a migratory endoparasitic pathogen, and it is difficult to use RNAi to silence genes in the wild. Second, the successful application of RNAi usually depends on effective gene silencing in target organisms. This study found that there were significant differences in the efficiency of gene silencing of PWNs after designing dsRNA in different regions of the gene. In this study, we used the dsRNA soaking method to silence the *Bx-SDR3* gene of PWNs, but further research is needed to explore more efficient and cost-effective delivery systems that could be applied at a larger scale. Additionally, the long-term effects of RNAi on PWN populations and potential non-target effects should be thoroughly investigated to assess the ecological risks and sustainability of this approach [26].

Nevertheless, our findings support the potential of RNAi as a valuable tool for improving the efficacy of chemical treatments like EB, offering a novel approach to enhance pest control while minimizing environmental impact. By combining RNAi-based strategies with traditional pest management techniques, it may be possible to develop integrated pest management (IPM) approaches that are both more efficient and environmentally friendly. Further research into the molecular biology of PWNs and the refinement of RNAi technology is crucial for translating these promising results into practical solutions for forest pest management.

## 4. Materials and Methods

### 4.1. Sample Collection and Processing

In this study, the strain FSBx of PWN was isolated from diseased *P. koraiensis* from Fushun and then purified and cultured. FSBx was cultured on *Botrytis cinerea* under dark conditions at 25 °C and subsequently propagated for further use. *P. koraiensis* was collected from Dahuofang Forest Farm, Fushun City, Liaoning Province, and one-year-old branches were used as experimental materials. The in vivo tests of PWNs involved in this paper were completed in the laboratory of the epidemic area-Shenyang Institute of Technology. After the completion of the test, the test materials and utensils involved in PWNs were harmlessly treated according to the requirements of relevant regulations.

### 4.2. The Effect of EB on the P. koraiensis Inoculated with PWNs

To evaluate the effect of EB on the number of PWN populations during PWN infection of *P. koraiensis*, EB was applied to *P. koraiensis* inoculated with PWNs, and the effect of EB was determined based on the symptoms of *P. koraiensis* and the number of isolated PWNs. The Berman funnel method was used to separate PWN from *B. cinerea*, and 50 PWNs/μL suspension was prepared.

The experimental group was inoculated with 100 μL of PWN suspension on one-year-old branches of *P. koraiensis*, cultured in water for 7 d, treated with 60 mg/L EB for 2 d, and then continued to culture in water for 11 d. CK1 was inoculated with 100 μL of PWN suspension by peeling the one-year-old branches of *P. koraiensis*, and then cultured in water for 20 d. CK2 was cultured with the same amount of sterile water on one-year-old branches of *P. koraiensis* for 20 d after simulated inoculation by ddH_2_O. Each group underwent three independent replicates. PWN in the branches of each treatment group were isolated every day and counted under an anatomical microscope, which was repeated three times.

To verify the content of EB in the branches of *P. koraiensis* following 2 d of EB treatment, targeted metabolomics was employed to quantify EB levels in the branches after 2 d of soaking. Each sample was analyzed with three biological replicates.

### 4.3. Mortality of PWNs by EB Treatment

To study the survival rate of PWN under different concentrations of EB and to explore whether PWN has a detoxification effect on EB, in vitro poisoning experiments of EB with five concentration gradients were carried out. EB solutions with different dilutions were added into 96-well plates, each with 100 microliters, and the final EB concentrations were 12 mg/L, 9 mg/L, 6 mg/L, 3 mg/L, and 1 mg/L. The control was represented by sterile water. Each concentration setup was reproduced five times. PWNs were added to each experimental group at the concentration of 50 nematodes per microliter. The culture plates were incubated in a dark environment at 25 °C, and the PWN mortality rate (MR) was recorded hourly over a 24-h period:

(1) $$\mathrm{MR}\;\left(\%\right)=\frac{n_{D}}{n_{A}}\times 100\%$$

where n*_D_* is number of dead nematodes and n_A_ is total number of nematodes.

The corrected mortality rate (CMR) was calculated as follows:

(2) $$\mathrm{CMR}\;(\%)=\frac{\mathrm{MR}1-\mathrm{MR}2}{100\%-\mathrm{MR}1}\times 100\%$$

where MR1 is the mortality rate from treatment group and MR2 is the mortality rate from CK.

Using the collected data, the toxicity regression equation was established, the corrected mortality rate was calculated, and LC_50_ and LC_20_ were determined. To verify the toxic effect of LC_20_, PWNs were treated with LC_20_ for 24 h, and the MR and CMR of PWNs were calculated. At the same time, the PWNs treated with LC_20_ for 24 h were stained with methyl blue for 10 min, and the staining solution was washed with M9 buffer. The dead nematodes could be dyed blue.

### 4.4. Transcriptomic Analysis

To reveal the reason why the PWN can still survive under low-dose conditions, we needed to look for genes that have the function of targeting EB detoxification through transcriptome detection of gene expression changes in PWN after agent treatment. The PWNs were exposed for 30 min and 5 h to LC_20_ EB and a CK that received a treatment of sterile water. After the treatment, PWNs were subjected to Trizol reagent for total RNA extraction. The RNA samples were sent to the Beijing Genomics Institute (BGI) for transcriptome sequencing. The differentially expressed genes (DEGs) were determined by analyzing gene expression levels that had been normalized, using a false discovery rate (FDR) threshold of less than 0.05 and |log_2_ fold change (LC_20_ EB/CK)| ≥ 1. KEGG Pathway enrichment analysis was performed on DEGs. Six genes were randomly selected to verify the reliability of transcriptome data by RT-qPCR (Primer sequence in Table S1).

### 4.5. WGCNA Analysis

To explore the function of detoxification-related genes of PWNs and further study the function of detoxification genes, the WGCNA was used for analysis. A co-expression network of detoxification genes in PWN was constructed using the WGCNA package (1.73) in R (4.1.3). Gene expression analysis was performed using Principal Component Analysis (PCA). Related modules were grouped together using a merge cut height of 0.25 to conduct cluster analysis.

### 4.6. Functional Enrichment Analysis of Candidate Module Genes

To study the function of selected detoxification-related genes and explore the role of the genes in key biological processes and metabolic pathways, 243 genes in the corresponding module were enriched by GO and KEGG. Potential modules consisting of substantially comparable genes were subjected to enrichment analysis and the cellular localization, biological functions, and pathway participation of these genes were determined by applying GO analysis. Key metabolic and signal transduction pathways were identified using KEGG analysis, and significant enrichment was determined for pathways with FDR ≤ 0.05 after correction for multiple testing. The transcriptome analysis identified *Bx-SDR3* as a candidate gene for further investigation.

### 4.7. Bx-SDR3 Gene Cloning and RNAi Experiment

Further verify the function of *Bx-SDR3* gene when PWN survived in low concentration EB (LC_20_), the gene was cloned and the function of *Bx-SDR3* gene was verified by RNAi. Total RNA was extracted from PWN and reverse transcription of cDNA was performed. According to the genome of PWN and the resequencing results of FSBx, specific primers were designed for gene cloning: *Bx-SDR3*-F sequence: 5′- ACA CGA ACG GAT TGT CAT CA -3′; *Bx-SDR3*-R sequence: 5′- GCT TTA GAC CTG TAG TGG ACA -3′. PCR products were sent to Sangon Biotech (Shanghai) Co., Ltd. (Chang chun, China) for sequencing. ORF analysis was performed using ORF-Finder. BLAST screened orthologous sequences collected by NCBI, Clustal W conducted multi-sequence alignment, and analyzed Bx-SDR3 conserved domain. The maximum likelihood tree (MLT) was constructed, and the branch support rate of the tree was obtained by the bootstrap method, with 1000 repeated sampling tests conducted.

Based on the complete reading frame sequence of *Bx-SDR3* gene, five segments of *Bx-SDR3* gene were designed with siRNA, and the segment with the best RNAi effect was screened through RNAi. The siRNA was labeled with carboxy fluorescein which interfered with *Bx-SDR3* gene separately (dsSDR300-1: 5′- GCA AAU UCA UUG GAU GGC UCC UUU A [dU] [dU] -3′; dsSDR300-2: 5′- CAG ACG CUU CCA AUC CCA UUG CCU A [dU] [dU] -3′; dsSDR300-3: 5′- UGC GGA AGA GUU UGA AUG GAA A [dU] [dU] -3′; dsSDR300-4: 5′- CGG AGU UCU GAA UGC GUC ACC GUU U [dU] [dU] -3′; 5′- CCA AGU UGA UCG AUG UGA AUA CGA A [dU] [dU] -3′), compared the *Bx-Ef-1α* gene RNAi cyanine dye labeled siRNA (5′- AGG AGC UGU UCA CCG GGG UGG UGC CCA UCC U [dT] [dT] -3′) and untargeted siRNA (5′- AGG AGC UGU UCA CCG GGG UGG UGC CCA UCC U [dT] [dT] -3′) performed RNAi interference test. The corresponding siRNA was diluted to 2 mg/mL (containing 10 mM octopamine) with diethylpyrocarbonate (DEPC)-treated ddH_2_O. After soaking for 12 h, images were taken using a fluorescence microscope. RNAi interference effects were identified by RT-qPCR (Primer Pairs: BXY-*SDR*-F: 5′- CTG GTG GAG GAA GGC TGT CAA -3′, BXY-*SDR*-R: 5′- ATT GTC TCG GTG TTG CTC TGG A -3′).

Dissolution curves were determined using 2-step PCR: 95 °C pre-denaturation for 2 min; 95 °C denaturation for 15 s, 60 °C annealing for 1 min, 40 reaction cycles, and 60–95 °C. *Bx-Ef-1α* (primer pair: *Bx-Ef-1α*-F: 5′- ATC GAC AAG CGT ACC ATC GAG -3′; *Bx-Ef-1α*-R: 5′- TAA TAC CAC GTT CAC GCT CA -3′) was used as a reference gene. The relative quantification method was employed to calculate the initial template quantity ratios across three replicates, and a significant difference between two paired samples was identified using a *t*-test.

The treatment group demonstrating the highest RNAi efficiency was designated as the *Bx-SDR3* RNAi group, while FSBX not subjected to RNAi treatment served as the control group. The *Bx-SDR3* RNAi group was immersed in dsRNA for 12 h, and the control group was immersed in DEPC-treated ddH_2_O for 12 h. Then the *Bx-SDR3* RNAi group and the control group were treated with LC_20_ concentration of EB, and the survival rate was counted after EB treatment for 24 h. Three independent replicates were performed at each time point to verify the function of the *Bx-SDR3* gene.

## 5. Conclusions

This study emphasized the potential of EB as a biological control agent for *P. koraiensis* in northeast China. While EB has proven effective in reducing PWN populations, the existence of detoxification mechanisms suggests that we need to refine our application techniques. Our research into the *Bx-SDR3* gene has yielded important insights into how PWNs detoxify. By using RNAi to target this gene, we have shown that PWNs become significantly more susceptible to low concentrations of EB. This innovative approach not only boosts the effectiveness of EB but also aligns with sustainable pest management practices. Overall, these findings play a vital role in the sustainable protection of valuable pine resources, reinforcing China’s commitment to ecological balance and the conservation of its rich natural heritage.

## Figures and Tables

**Table 1:** Toxicity of EB with different concentrations to PWNs.

Concentration (mg/L)	Mortality Rate	Corrected Mortality Rate	Virulence Regression Curve	95% Confidence Limit of LC_50_
12	100%	100%		
9	(95.02 ± 0.026)%	(95.01 ± 0.026)%		
6	(67.33 ± 0.025)%	(67.26 ± 0.026)%	Y = 1.289 + 2.366x	0.046~0.586
3	(49.48 ± 0.041)%	(49.37 ± 0.042)%		
1	(21.19 ± 0.031)%	(21.03 ± 0.030)%		

## Data Availability

Data are contained within the article or Supplementary Materials. The original contributions presented in this study are included in the article/Supplementary Material. Further inquiries can be directed to the corresponding author.

## Supplementary Materials

The following supporting information can be downloaded at: https://www.mdpi.com/article/10.3390/ijms26041679/s1.

## Abbreviations

The following abbreviations are used in this manuscript:

PWD	pine wilt disease
PWN	pine wood nematodes
*P. koraiensis*	*Pinus koraiensis*
EB	emamectin benzoate
SDR	Short-chain dehydrogenases/reductase
RNAi	RNA interference
*H**. contortus*	*Haemonchus contortus*
LC_20_	the concentration at 20%
CK2	the control group 2
CK1	the control group 1
LC_50_	the half lethal concentration
KEGG	Kyoto Encyclopedia of Genes and Genomes
WGCNA	weighted gene co-expression network analysis
GO	Gene Ontology
Ala	alanine
Asp	aspartate
Glu	glutamate
Gly	glycine
Ser	serine
Thr	threonine
ORF	open reading frame
NCBI	National Center for Biotechnology Information
IC	inhibitory concentration
*C. elegans*	*Caenorhabditis elegans*
FMO	flavin-containing monooxygenase
MR	mortality rate
CMR	corrected mortality rate
BGI	Beijing Genomics Institute
DEGs	differentially expressed genes
FDR	false discovery rate
PCA	principal component analysis
MLT	maximum likelihood tree
DEPC	diethylpyrocarbonate
