# Supplementary material for: Effective Dose Reduction of Emamectin Benzoate Through Inhibition of Bx-SDR3 in Pine Wood Nematode Management
Source: Int J Mol Sci. 2025 Feb 16;26(4):1679. doi: 10.3390/ijms26041679 (PMC11855115; doi:10.3390/ijms26041679)
Supplement: Supplementary file 1 [file ijms-26-01679-s001.zip › ijms-3456429-supplementary.pdf]

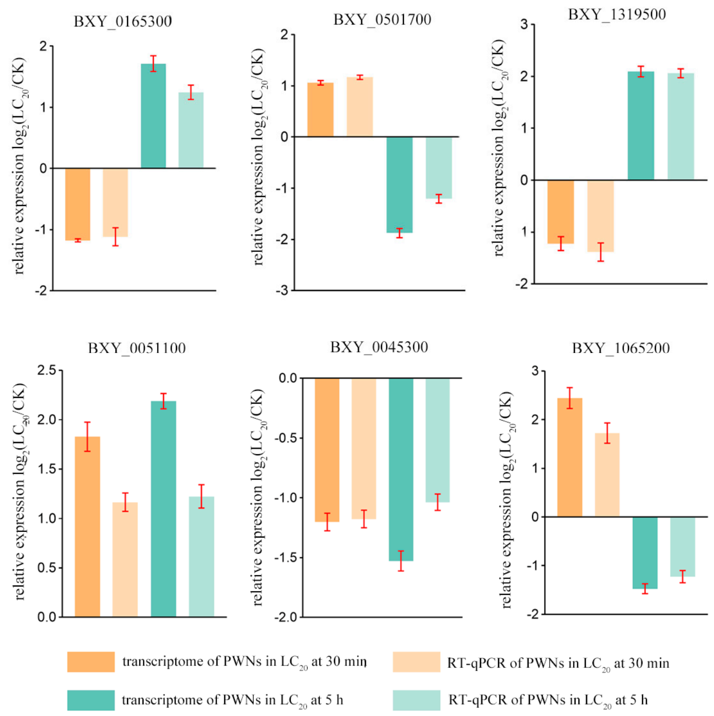

Figure S1. RT-qPCR verification of DEG in the transcriptome.

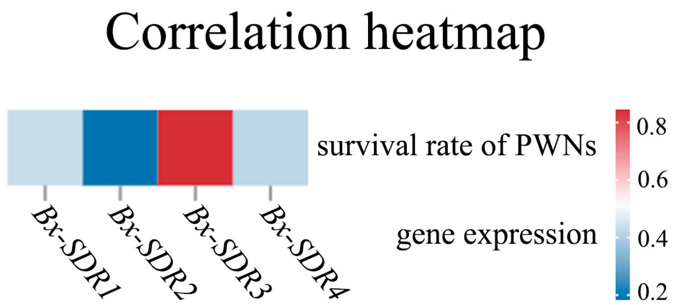

Figure S2. Correlation analysis between Bx-SDR gene expression and survival rate of PWNs.

Table S1. RT–qPCR Primers used in this experiment.

Gene	Forward Primer	Reverse Primer
BXY_0165300	CCGAAACTGAACTCCGAGA	CCTTGAGAGCTTTCTTGGCT
BXY_0045300	AGCCAACAACTCATCCAGAA	CTCAGCGCAATCAACGTTTC
BXY_0051100	ATTTCGGCCATCTTCTTGCT	TGATGCAGCGTAGCTTATCG
BXY_1319500	AAATCGTACTTCTCGTTTGCG	ACTGAAACCGTTCCATTTGC
BXY_0501700	GGAATGGCGTTTCTTGAGATG	CAAGGTGAAGGTGTCAGCAT
BXY_1065200	GAATGCTCGCCCACCTC	TTGAACCATGTCAAACCCGA
